# A single bisphosphonate infusion does not accelerate fracture healing in high tibial osteotomies: A randomized study of 46 patients

**DOI:** 10.3109/17453674.2011.594231

**Published:** 2011-09-02

**Authors:** Anna Kajsa Harding, Annette W-Dahl, Mats Geijer, Sören Toksvig-Larsen, Magnus Tägil

**Affiliations:** ^1^Department of Orthopedics, Clinical Sciences, Lund University and Skåne University Hospital, Lund; ^2^Department of Orthopedics, Hässleholm Hospital, Hässleholm; ^3^Centre for medical imaging and physiology, Skåne University Hospital, Lund, Sweden; Correspondence: Anna_Kajsa.Harding@med.lu.se

## Abstract

**Background:**

Bisphosphonates increase the callus size and strength in animal fracture studies. In a human non-randomized pilot study of high tibial osteotomies in knee osteoarthritis, using the hemicallotasis (HCO) technique, bisphosphonates shortened the healing time by 12 days. In the present randomized study, we wanted to determine whether a single infusion of zoledronic acid reduces the time to clinical osteotomy healing. Results from the same trial, showing improved pin fixation with zoledronate, have been published separately.

**Methods:**

46 consecutive patients (aged 35–65 years) were operated. At 4 weeks postoperatively, the patients were randomized to an intravenous infusion of either zoledronic acid or sodium chloride. Dual-energy X-ray absorptiometry (DEXA) was performed 10 weeks postoperatively. Radiographs were taken at 10 weeks and every second week until there was radiographic and clinical healing. Healing was evaluated blind, with extraction of the external fixator as the endpoint. At 1.5 years, an additional radiograph was taken and the hip-knee-ankle (HKA) angle measured to evaluate whether correction had been retained.

**Results:**

All osteotomies healed with no difference in healing time between the groups (77 (SD 7) days). Bone mineral density and bone mineral content, as assessed with DEXA, were similar between the groups. Radiographically, both groups had retained the acquired correction at the 1.5-year follow-up.

**Interpretation:**

In this randomized comparison, a single infusion of zoledronic acid increased the pin fixation of the external frame but did not shorten the healing time. In both groups, the external fixator was extracted almost 2 weeks earlier than in previous studies. The early extraction did not cause a loss of correction in either group.

Pharmaceutical substances, both anabolic and anti-catabolic, have been used to modulate fracture healing (for a review, see Aspenberg 2005). Anabolic drugs such as parathyroid hormone (PTH) or bone morphogenic protein (BMP) influence the osteoblasts, either unspecifically by increasing recruitment and proliferation, or specifically by directing the progenitor cells into the osteoblastic lineage (Schindeler et al. 2008). Anti-catabolic drugs such as bisphosphonates reduce the osteoclastic activity, and have been shown to increase the strength of a healing fracture by retaining the new-formed callus (Amanat et al. 2007).

Both anabolic and anti-catabolic drugs can therefore affect fracture healing, but the efficiency and mode of action of these drugs appear to differ between open and closed fractures (Tägil et al. 2010). In open fractures and other fractures associated with delayed union like distal tibial fractures, a specific anabolic drug such as a BMP sometimes appears to be necessary (Little et al. 2005, Ristiniemi et al. 2007). In a fracture in a cell-rich, well-vascularized environment an anti-catabolic drug might be sufficient (Amanat et al. 2007). Several arguments can be put forward for both the clinician and the patient to use an anti-catabolic drug instead of an anabolic one for these fractures. Apart from being considerably more expensive, the existing anabolic drugs have to be delivered into the fracture gap directly (BMP) or injected daily during the healing time (PTH).

In this study, we wanted to determine whether an anti-catabolic drug would reduce the time to healing in a well-vascularized fracture, using HCO in knee osteoarthritis as a relatively standardized and relatively frequent human model of bone healing. In a non-randomized pilot study of 24 patients operated on by HCO, 12 patients were treated with one single infusion of zoledronic acid and 12 were not. In the treatment group, the frame was removed after 78 (SD 13) days as compared to 91 (SD 13) days in the untreated group (p = 0.02) (Tägil et al. 2006). In a previous study from the same group studying the influence of smoking, the treatment time in external fixator was 96 days in non-smoking patients (W-Dahl and Toksvig-Larsen 2004).

In the present randomized study, the primary outcome was clinical fracture healing, evaluated blind, and we wanted to determine whether one single infusion of zoledronic acid could reduce the time to fracture healing. As secondary outcome, we evaluated the pin fixation and found almost 100% improvement in pin fixation in the uncoated pin in diaphyseal bone (Harding et al. 2010). Furthermore, the bone mineral density and bone mineral content of the osteotomy gap were compared between groups to monitor the biological effect of the single dose. Drug safety was evaluated, as well as the subjective outcome as measured with the knee-specific KOOS questionnaire. Finally, we examined whether the surgically achieved correction was maintained, using radiographic hip-knee-ankle (HKA) angle measurement after 1.5 years.

## Patients and methods

**Figure 1.**
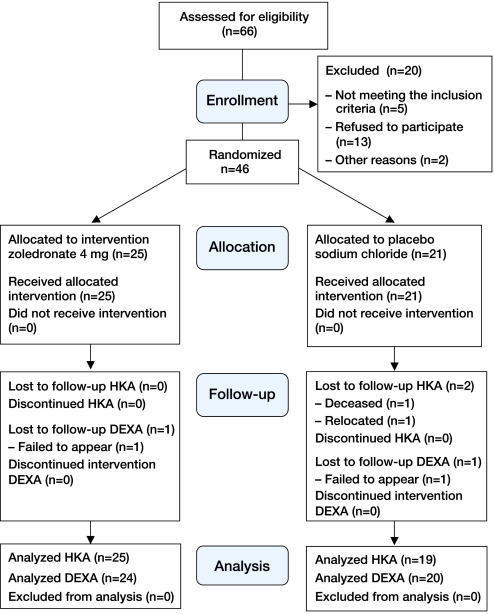
A CONSORT flow diagram depicting patient recruitment, randomization, patient flow, and follow-up in the study.

Between February 2006 and May 2007, 46 patients (36 men) with a mean age of 49 (37–63) years (Table 1) who were operated on for knee osteoarthritis by the hemicallotasis technique (HCO) were included in the study (Figure 1). 41 patients had medial compartment osteoarthritis and 5 patients had lateral compartment osteoarthritis. Inclusion criteria were age between 35 and 65 years and osteoarthritis or deformity of the knee requiring surgery. Exclusion criteria were kidney, liver, or odontological disorders; rheumatoid arthritis; or bisphosphonate treatment two years or less before surgery. Preoperatively, the patients were given written and oral information and informed consent was obtained. The study was performed in compliance with the Helsinki Declaration and was approved by both the local ethics committee of Lund University (2006-03-02; registration number 554/2005) and the Swedish Medical Product Agency, and was registered in the EUDRACT database. It was externally monitored by a hospital-based but independent organization (RSKC, Lund University Hospital). All patients who met the inclusion criteria were prescribed daily oral calcium and vitamin D3 the first 6 weeks postoperatively, to avoid hypocalcemia if given the active drug.

**Table 1.** Patient characteristics

	Zoledronate group (n = 25)	Control group (n = 21)
Men/women	19/6	17/4
Mean age in years (SD)	48 (7)	50 (5)
Mean pre-HKA in degrees (SD)	173 (6)	173 (5)
Mean BMI (SD)	28 (3)	26 (3)
Smoker	4	7
Unsmoked tobacco user	4	6

HKA: hip-knee-ankle angle (< 180° = varus).

### Surgery and randomization

**Figure 2.**
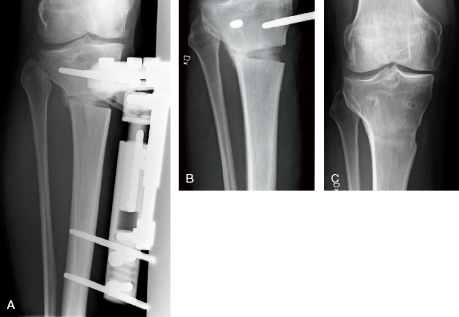
Hemicallotasis osteotomy using the Orthofix T-garche as external fixator. A. Successive lengthening takes place. The HKA angle is slowly normalized and the frame is locked at the desired angle. The frame is kept until bone healing. B. When sufficient callus appears to be present by ultrasound and radiography, the frame is removed temporarily. The patient is allowed to bear weight before the final decision to remove the frame. This decision is made blind regarding the pharmacological treatment. C. Radiograph showing a healed osteotomy at 1.5 years.

The HCO was performed as an outpatient procedure using the Orthofix T-garche external fixator (Magyar et al. 1998) (Figure 2). 4 conical stainless steel pins were inserted: 2 hydroxyapatite- (HA-) coated pins (Orthofix, Bussolengo, Italy) in the metaphyseal bone and 2 uncoated pins (Orthofix) in the diaphyseal bone. The patients were allowed free mobilization and full weight bearing after the operation. The distraction started 7–10 days postoperatively. The patient made the correction by adjusting a distractor placed at the external fixator, one quarter of a turn 4 times per day, with the first turn in the morning and the last turn in the afternoon. Correction was measured by the hip-knee-ankle (HKA) angle of the knee during the corrective phase until the desired correction was achieved—4° valgus for the varus knee and 0–2° varus for the valgus knee—and the instrument was locked. The HKA angle was measured radiographically on an anteroposterior view of the lower limb (hip, knee, and ankle) with the patient standing and weight bearing. By drawing a line from the center of the femoral head to the midpoint of the spine of the tibial eminence and another line from this midpoint to the center of the talus surface of the ankle joint, the mechanical axis of the limb could be calculated (Siu et al. 1991). The medial angle between the lines is the HKA angle (varus < 180°). The accuracy and reproducibility of measurement of the HKA angle has been shown to be within 2° (Odenbring et al. 1993).

4 weeks postoperatively, randomization was performed by one of the authors (AWD) using closed envelopes. The patient received an infusion of either zoledronic acid (4 mg intravenously) (Zometa; Novartis Pharma GmbH, Basel, Switzerland) or sodium chloride (9 mg/mL) prepared by the unblinded nurse. The infusion of zoledronic acid was prepared by diluting 4 mg zoledronic acid in 100 mL sodium chloride (9 mg/mL) and the zoledronic acid or saline solution was given as a 15-min intravenous infusion. The patients were blinded regarding the type of infusion. If a patient reported signs of hypocalcemia at the follow up, serum calcium was measured.

### Outcome: Time to healing

At 8 weeks postoperatively, radiographs of the lower leg were obtained without weight bearing, and at 10 weeks the first evaluation of healing, blinded, on radiographs and ultrasound was done. The ultrasonic examination shows unmineralized callus formation in the distracted area earlier than conventional radiographs. When the callus was judged to be sufficient by radiographic examination (consolidation of about two-thirds of the osteotomy gap) and ultrasonic examination, the patient did a weight-bearing test, i.e. walking without the fixator but the pins still in situ for 2–48 hours. If the patient reported symptoms of incomplete healing, mainly pain by loading, the fixator was re-applied for 2 additional weeks (Table 2). The patient was checked radiographically every second week, and by ultrasound examination—until healing of the bone, again under blinding, was satisfactory (Magyar et al. 1998).

**Table 2.** Number of patients who were healed at each evaluation time point

Week	Zoledronate group (n = 25)	Control group (n = 21)	Total (n = 46)
10	17	18	35
12	24	20	44
14	24	21	45
16	25	21	46

### KOOS (clinical outcome)

The form for the patient-relevant outcome measure (KOOS) (Roos et al. 1998a, b) was filled in preoperatively, and at 8 and 10 weeks and every second week postoperatively, until extraction of the external fixation. It was also filled in at the 20-week and 1.5-year follow-up.

### Evaluation of densitometry

Dual-energy X-ray absorptiometry (DEXA) of the operated lower leg and bilateral proximal femur was performed at 10 weeks postoperatively. The fixator was removed but the pins were still in place. The DEXA scans were centered anteroposteriorly over the osteotomy and 2–3 scans were performed to avoid over-projection of the fibula. Only 1 of the scans was used for evaluation. The region of interest (ROI) was centered over the osteotomy, including the edges. When the osteotomy gap was almost consolidated at the DEXA images, the borders of the osteotomy were measured on the corresponding radiograph and transferred to the DEXA image. The bone mineral density (BMD) and the bone mineral content (BMC) were measured. The total T-score of the proximal femur was used to determine whether the patients had osteoporosis (< –2.5 SD), osteopenia (–1 to –2.5 SD), or normal bone. All scans were performed using GE Lunar Prodigy 16196 (Lunar BMP Products, Madison, WI).

### Evaluation of the retention of surgically achieved correction

Radiographs were taken at mean 20 (SD 2) months after surgery, with measurement of the HKA angle to determine whether the correction was retained. All radiographs were analyzed and measured by one radiologist (MG). The value of the HKA angle was compared to an estimated angle of the correction, i.e. 4° valgus for the varus knee and 0–2° varus for the valgus knee. This was used as a measurement of the retention of correction.

### Safety

All drug-related side effects and adverse events were registered prospectively during the study. Complications such as delayed healing (by definition, > 16 weeks in external fixation (W-Dahl and Toksvig-Larsen 2004)), non-union, septic arthritis, deep venous thrombosis, loss of correction, etc. (including replacement of pins and difficulties in correction) were recorded.

### Statistics

A power analysis was performed based on the data from the pilot study. 25 samples would be needed in each group to achieve a power of 95% at p = 0.05 in a two-sided test, with an estimated mean difference of healing time of 20 days between the bisphosphonate group and the control group. All data were expressed as mean, and standard deviation of the mean or 95% confidence interval (CI). Student's t test was used to test the differences between the groups, with p < 0.05 being considered statistically significant in a 2-sided test. Statistical analysis was carried out using SPSS software version 16.0.

## Results

### Surgery

All osteotomies healed, and the time in external fixation was the same in both groups: 77 (95% CI: 75–80) days in the zoledronic-treated group and 77 (CI: 74–81) days in the control group. The mean difference was 0.2 (CI: –4.4 to 4.8) days. 17 of 25 patients in the zoledronic group and 18 of 21 patients in the control group had healed after 10 weeks in external fixation (Table 2). No patients had signs of hypocalcemia.

### KOOS

The KOOS score improved over time. The major improvements in pain, symptoms, ADL, and QOL were already achieved during treatment in the external fixator, whereas for sports and recreation the improvements could be measured first after the external fixator had been removed. There were only small differences between the groups, none of themwhich were statistically significant.

### DEXA

2 of the patients were lost to follow–up by DEXA (n = 44). None of the remaining patients had osteoporosis. 3 patients in the zoledronic-treated group and 6 in the control group had osteopenia. BMD of the healing tissue in the osteotomy gap was 1.14 (SD 0.27) g/cm² in the zoledronic treated group, as compared to 1.01 (SD 0.18) g/cm² in the control group (p = 0.1). BMC of the osteotomy gap was 6.2 (SD 2.3) g in the zoledronic treated and 5.2 (SD 2.3) g in the control group (p = 0.2).

### HKA

2 patients were lost to follow-up at 1.5 years after surgery (n = 44). No difference were seen in the change of HKA angle between the groups. In both groups, the changes were less than the measurement error of 2 degrees: the mean change was 0.3 (SD 3.3) degrees in the zoledronic-treated group as compared to –1.0 (SD 3.3) degrees in the control group (p = 0.2). When the patients with lateral knee osteoarthritis were excluded, the mean difference was 0.5 (SD 3.4) degrees in the zoledronic-treated group and –1.1 (SD 3.6) degrees in the control group (p = 0.2).

### Safety

No difference was found between the 2 groups regarding complications. One deep vein thrombosis was observed in the control group. 13 of 25 patients reported muscle pain and influenza-like symptoms in the zoledronic group as compared to 2 of 21 in the placebo group (RR = 5, CI: 1.3–20; p = 0.004). One serious adverse event was registered in the zoledronic group, in the form of chest pain due to pneumonia.

## Discussion

In a non-randomized pilot study of 24 patients, operated on using the HCO technique, 12 patients were treated with a single infusion of zoledronic acid and 12 were not (Tägil et al. 2006). In that study, the external fixator was removed after mean 78 (SD 13) days in the treated group as compared to 91 (SD 13) days in the untreated group (p = 0.02). In the present randomized study, the mean healing time was 77 days in both groups, i.e. shorter than in the pilot study but also shorter than in a previous study, in which the mean healing time was 96 days (W-Dahl and Toksvig-Larsen 2004). The follow-up and the methods used to evaluate healing were the same as in the previous studies from our group (W-Dahl and Toksvig-Larsen 2004, 2008), and in all of them there was blinding as to treatment. A true shortening of the treatment time therefore occurred in this study, but this was unrelated to administration of the drug. A fear that extraction of the external fixator too early would cause a loss of the surgically achieved HKA correction could not be verified at the late radiographic control. No loss of correction was found in either group 1.5 years after surgery, and the values measured were within the measurement error.

With the time from surgery to extraction shortened in both groups, it appears that the experimental protocol was inappropriate for finding differences. The first evaluation of healing was done after 10 weeks, i.e. a time point close to the shortened clinical healing time in both groups. Also, the DEXA scans were performed at 10 weeks after surgery and at this time point many of the osteotomies in both groups were healed. Perhaps an earlier time point for evaluation of the effect of the drug should have been used, but the time from administration of the drug to extraction would then have been less than 6 weeks, which at the time of design of the study was considered the minimum time for the drug to have an effect.

The choice of time point for administration of the drug was based on animal experiments (Amanat et al. 2007). Bisphosphonates bind to bone mineral and if given at the time of on-going fracture healing, a larger proportion binds to the fracture site than if given earlier, for example at the time of the osteotomy. In animals, delayed administration of the bisphosphonate has been shown to have a superior effect compared both to saline and compared to zoledronic acid administered immediately at the time of the fracture (Amanat et al. 2007). A second reason for administration of the bisphosphonate at 4 weeks was to avoid disturbing the initial bone formation, which has been feared both in in-vitro studies (Im et al. 2004) and, perhaps more importantly, in in-vivo studies (Jakobsen et al. 2007).

2 other randomized studies have evaluated a drug regarding time to healing for a fracture in well-vascularized metaphyseal bone. PTH given as a daily injection was shown to reduce the time to healing in distal radial fractures by 1.7 weeks in a low-dose group (20 μg) but not in a high-dose group (Aspenberg et al. 2010). In a similar study comparing simvastatin and saline, also in distal radial fractures, no difference was found between the active compound and the control (Patil et al. 2009). Although both of these studies were in closed fractures, in contrast to ours, which was made in surgically opened fractures; we believe that the studies are comparable. In our study, the osteotomy was performed in well-vascularized metaphyseal bone where the access to circulation and cells is high. It resembles a closed-fracture animal model and there is no need to bolster the anabolic part of the fracture healing, as in an open fracture with compromised bone formation.

Based on the experience from animal experiments (Amanat et al. 2007, Harding et al. 2008), we used a potent bisphosphonate, zoledronic acid, which was administered as a single infusion 4 weeks after surgery. The dose was 4 mg, which was the dose used in treatment of malignancies at the time of design of the study, whereas the dose in osteoporosis today is 5 mg. Zoledronate was not approved for osteoporosis at the time this trial started. No difference was found in BMD or BMC between the groups, which may indicate that a single dose, although efficient in animals, might be insufficient in humans. Perhaps a higher dose would have shortened the healing time, but the risk of side effects and unwanted effects such as local toxic effects to the osteoblasts would be higher. It is possible that continuous administration of a weekly oral dose of a bisphosphonate might have been more effective, but it is also possible that there is simply no or a very limited effect of the drug in this model. Given the reputation of the drug, which is feared to be a negative modulator in fracture healing, it should be emphasized that no negative effects of the drug regarding bone healing were found.
